# Application of the “半” shaped crown sectioning method in minimally invasive extraction of mandibular horizontally impacted teeth

**DOI:** 10.1097/MD.0000000000042531

**Published:** 2025-05-16

**Authors:** Caiwang Chang, Le Yang, Juanjuan Gao, Miaomiao Shao, Qingmei Kong, Tingting Zhang, Jinhua Huang, Zhibing Meng

**Affiliations:** a Department of Oral and Maxillofacial Surgery, The Affiliated Hospital of Yangzhou University, Yangzhou University, Yangzhou, Jiangsu, P.R. China.

**Keywords:** “半” shaped crown sectioning method, crown segmentation method, extraction time, mandibular horizontally impacted teeth, micro-power system, minimally invasive surgery, tooth extraction, VAS score

## Abstract

Mandibular horizontally impacted horizontally impacted teeth (MHIT) often require extraction due to complications such as pericoronitis, caries, and periodontal disease. This study explores the efficacy of the “半” shaped crown sectioning method for these extractions. From May 2021 to May 2023, 240 patients undergoing extraction of MHIT were randomly and double-blindly assigned to the study group. (n = 120) using a micro-power system or the control group (n = 120) using an ultrasonic instrument. Key outcomes measured included operation time, alveolar damage, postoperative pain (VAS), mouth opening degree, and wound healing. The study group showed shorter operation times, lower alveolar damage scores, lower VAS scores, greater mouth opening degrees, and better wound healing than the control group (*P* < .05). The “半” shaped crown sectioning method significantly reduces buccal alveolar bone removal, operation time, alveolar damage, and postoperative pain while improving mouth opening and wound healing, demonstrating substantial clinical value.

## 1. Introduction

Impacted mandibular third molars are common in dentistry, typically manifesting as dysfunction of adjacent teeth, bone, or surrounding soft tissues, preventing the teeth from fully or partially erupting and obstructing future successful eruptions.^[1]^ MHITs, in particular, are significant contributors to oral diseases such as dental caries and pericoronitis in adjacent teeth and may lead to severe complications such as jaw osteomyelitis and oral space infections.^[2,3]^ The extraction of impacted teeth is one of the most technically challenging and complex procedures in oral surgery, carrying a high risk of postoperative complications.^[4]^

Traditional turbine-driven handpieces usually involve horizontally sectioning the tooth crown, removing surrounding bone, and creating space with conventional instruments to extract the impacted tooth. While this method is easy to master and widely adopted, it is time-consuming and highly traumatic. Limited operative space around impacted teeth sometimes necessitates incising the distal gingiva and removing distal and buccal surrounding bone tissue to expand the operative field. This can prolong postoperative healing time and increase the risk of complications, potentially affecting the patient’s quality of life post-surgery, which contradicts the concept of “minimally invasive surgery.”^[5]^

In contrast, the “半” shaped crown sectioning method using a micro-power system is feasible. This method sequentially sections the tooth crown, primarily by breaking down the crown to eliminate resistance from adjacent teeth, surrounding bone tissue, and inter-radicular areas. This facilitates the extraction of impacted teeth while minimizing trauma to the surrounding tissues. Additionally, the use of low-temperature sterile saline in combination with a micro-power system for systematic operation allows for the phased removal of all tooth tissue by leveraging internal and interstitial tooth structures as support. This approach effectively reduces or eliminates the need for repeated hammering and multiple elevations, thereby shortening operation time, reducing patient psychological stress, minimizing trauma, and alleviating postoperative swelling and pain.

This study aims to explore the application effects of the “半” shaped crown sectioning method in MHIT extraction to evaluate its clinical value.

### 1.1. Scientific contribution

This study provides a detailed evaluation of the “半” shaped crown sectioning method using a micro-power system to extract MHIT. By comparing this method with the traditional technique, the research aims to demonstrate improvements in operation time, trauma level, and postoperative recovery. The findings are intended to enhance current surgical practices by promoting minimally invasive techniques that could improve patient outcomes and reduce complications associated with impacted tooth extraction.

### 1.2. Scientific hypotheses

The “半” shaped crown sectioning method using a micro-power system will significantly reduce the operation time required for the extraction of MHIT compared to the traditional technique.

The “半” shaped crown sectioning method will cause less trauma to the surrounding tissues, resulting in a faster postoperative healing process compared to the traditional method.

Patients undergoing the “半” shaped crown sectioning method will experience reduced postoperative pain and fewer complications, improving overall quality of life post-surgery.

The findings are reported as follows.

## 2. Materials and methods

### 2.1. General information

**Figure 1.**
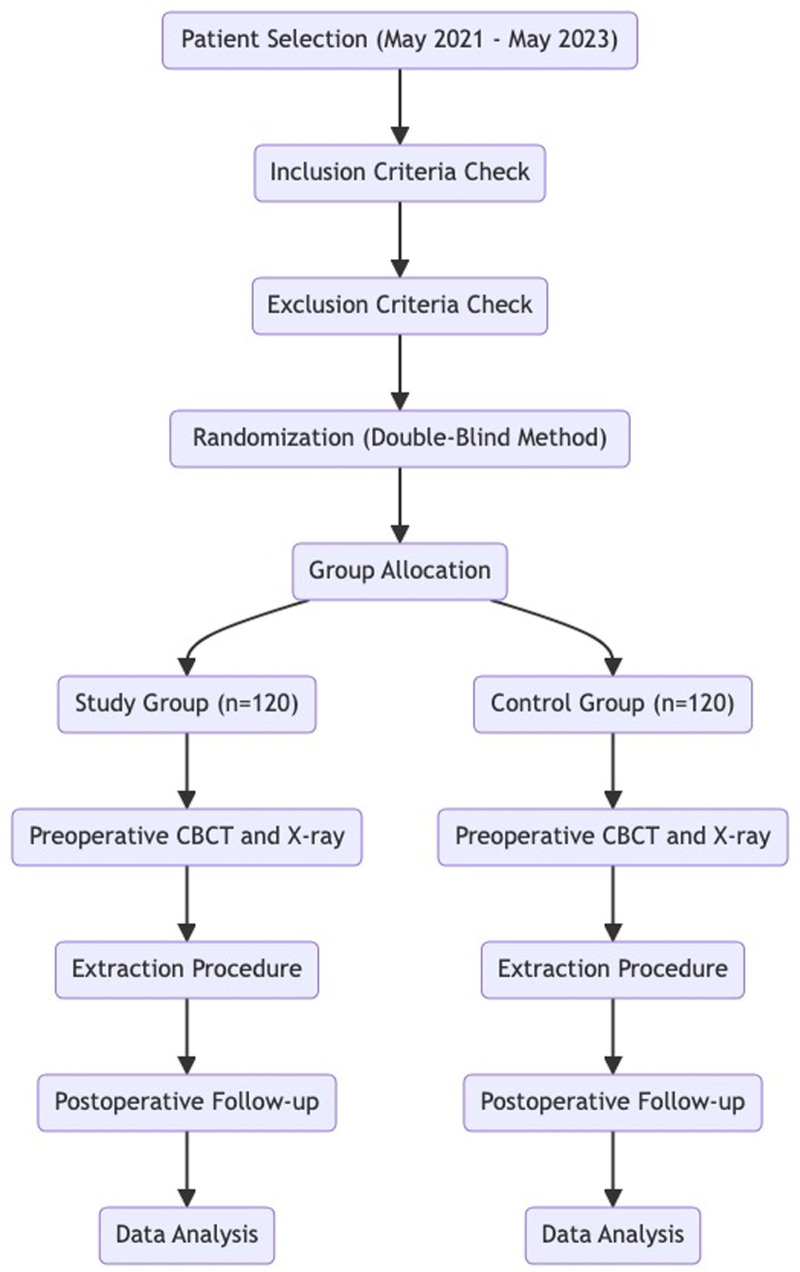
Study flowchart. This flowchart illustrates the process of patient selection and allocation for the study conducted between May 2021 and May 2023. Patients were selected based on specific inclusion and exclusion criteria. Following a double-blind randomization method, 240 patients were equally divided into 2 groups: the study group and the control group, each consisting of 120 patients. Both groups underwent preoperative CBCT and X-ray examinations followed by the extraction procedure. Postoperative follow-up was conducted for both groups, and data were analyzed to compare the outcomes. CBCT = cone-beam computed tomography.

From May 2021 to May 2023, 240 patients who underwent extraction of MHIT in our hospital’s Department of Stomatology were included in the study (Fig. 1).

Ethical approval and consent to participate: All patients signed informed consent forms, and the study was approved by the ethics committee of The Affiliated Hospital of Yangzhou University. The ethics committee waived the need for additional consent for this study as all procedures were part of routine clinical practice.

Inclusion criteria:

- Met the diagnostic criteria in “Transalveolar Extraction of the Mandibular Third Molars.”^[6]^
- Age ≥ 18 years.
- Eligible for tooth extraction surgery with no contraindications.
- No underlying diseases.
- Surrounding teeth are free from dental caries and periodontal disease.
- No inflammation or pain response in the affected tooth area before surgery.

Exclusion criteria:

- Poor compliance.
- Refusal to undergo preoperative cone-beam computed tomography (CBCT) and X-ray examinations.
- Lost to follow-up.
- Combined with other oral diseases.

Using a randomized double-blind method, the patients were divided into a study group (n = 120) and a control group (n = 120).

In the study group, there were 62 males and 58 females, aged 19 to 55 years, with an average age of (33.44 ± 5.45) years; 58 had a smoking history, and 70 had a history of alcohol consumption. In the control group, there were 61 males and 59 females, aged 20 to 56 years, with an average age of (34.98 ± 5.55) years; 53 had a smoking history, and 78 had a history of alcohol consumption. There were no significant differences in baseline data between the 2 groups (*P* > .05).

### 2.2. Universal methodology presentation

Figure 1 outlines the universal methodology used in this study.

### 2.3. Treatment methods

#### 2.3.1. *Preparation*

- Disinfect the micro-power system and ultrasonic instrument, and prepare the burs and handpiece. This step ensures a sterile environment and prevents postoperative infections.
- Prepare the saliva suction equipment. Saliva control is essential to maintain a clear operative field.
- Take panoramic or CBCT images of the patient’s mouth. This is crucial for assessing the peripheral resistance of the impacted tooth and its relationship with important surrounding anatomical structures, enabling precise planning of the extraction procedure.
- Fully communicate with the patient and have them sign the informed consent form. Obtaining informed consent ensures that the patient is aware of the procedure, risks, and benefits, complying with ethical standards.

#### 2.3.2. *Anesthesia*

- Disinfect the area of the affected tooth with 0.5% iodine. This reduces the risk of infection.
- Use 2 mL of 2% lidocaine hydrochloride injection for inferior alveolar nerve block anesthesia. Lidocaine is chosen for its efficacy and safety in providing the deep anesthesia required for this procedure.
- Perform local infiltration anesthesia with 1 mL of articaine adrenaline injection in the retromolar and buccal areas. Articaine provides effective local anesthesia with added vasoconstrictive properties from adrenaline, reducing bleeding during surgery.

#### 2.3.3. Surgical procedures

##### 2.3.3.1. Control group (ultrasonic instrument cutting method)

- Ensure satisfactory anesthesia to achieve pain control and patient comfort.
- Separate the gingiva and design a conventional angular flap incision. This provides adequate access to the impacted tooth.
- Remove the bone tissue covering the impacted tooth to expose the tooth crown. Necessary for visual and instrument access to the tooth.
- Use an ultrasonic bone surgery tip to cut the neck of the tooth at a 45° angle from buccal to lingual direction.
- Remove part of the adjacent buccal bone tissue. This step facilitates easier tooth removal by reducing mechanical resistance.
- Extract the crown first, then the root. Sequential extraction minimizes the risk of tooth fracture and ensures complete removal.

##### 2.3.3.2. Study group (“半” shaped crown sectioning method)

**Figure 2.**
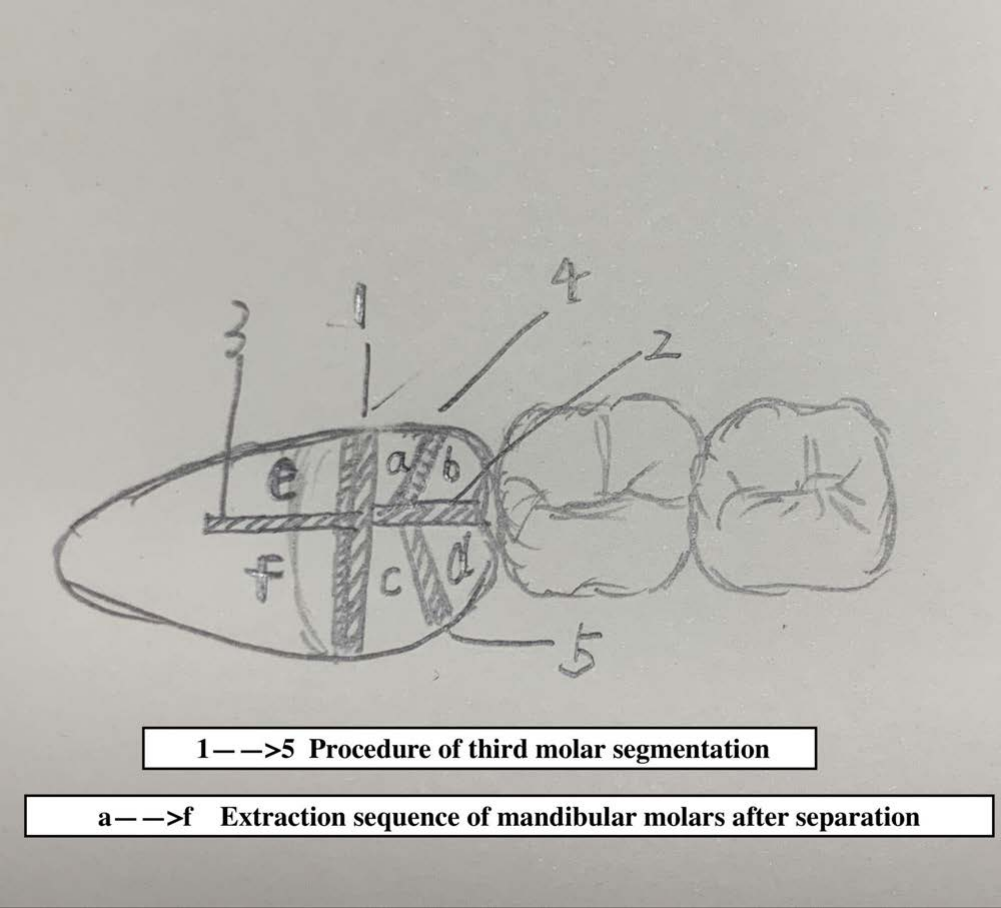
Diagram illustrating the sequence of sectioning and extraction of impacted teeth.

**Figure 3.**
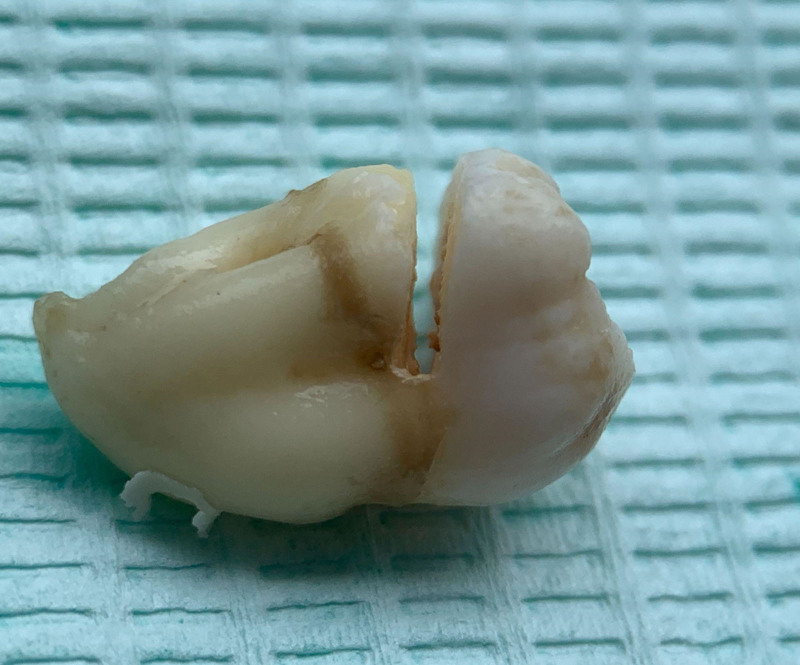
Buccal view depicts the separation of the crown and root from the buccal aspect.

**Figure 4.**
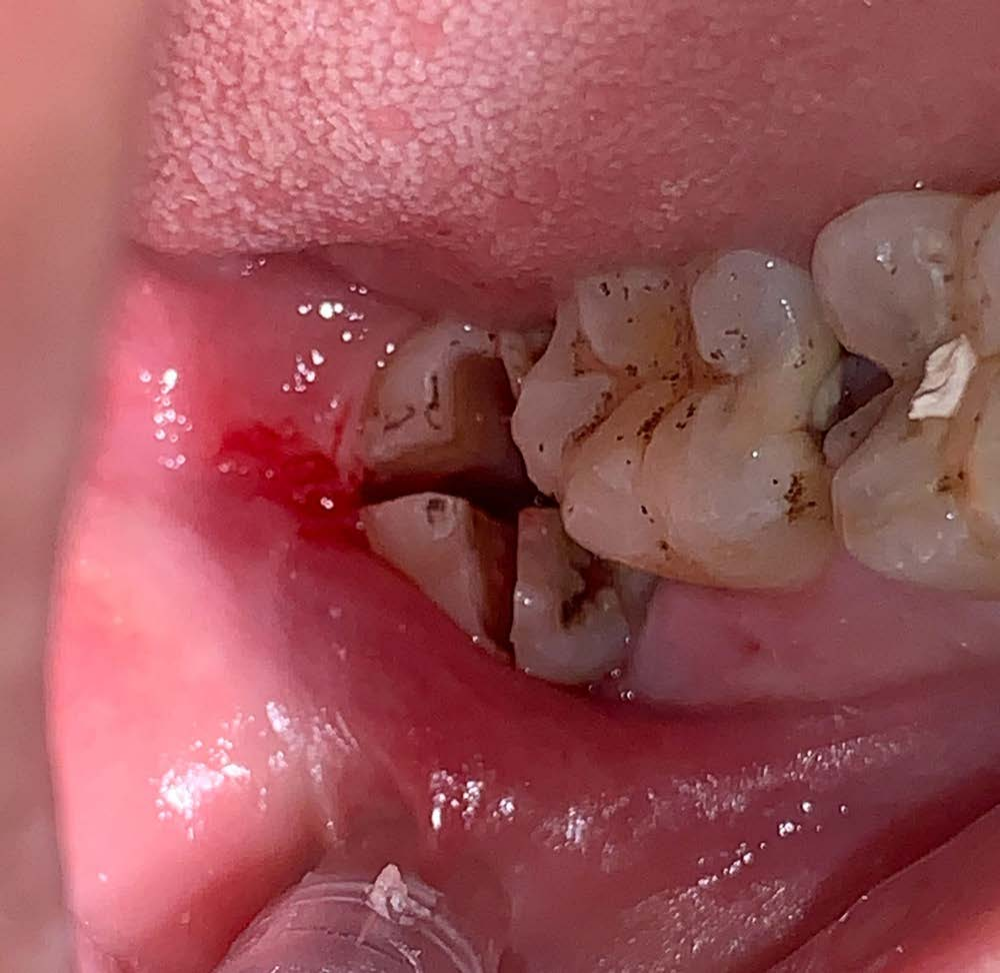
Occlusal view shows the occlusal surface with the corresponding MHIT segmentation. MHIT = mandibular horizontally impacted teeth.

**Figure 5.**
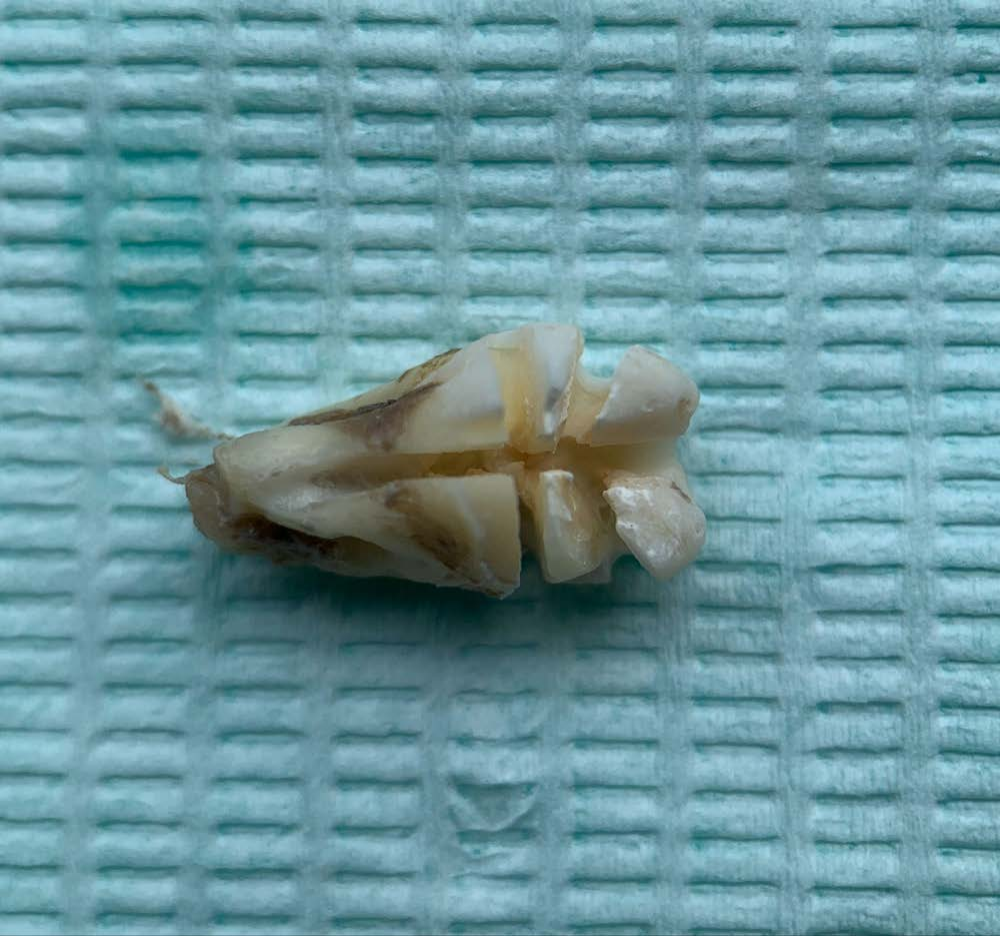
Buccal view shows final separation: illustrates the completion of the separation process.

**Figure 6.**
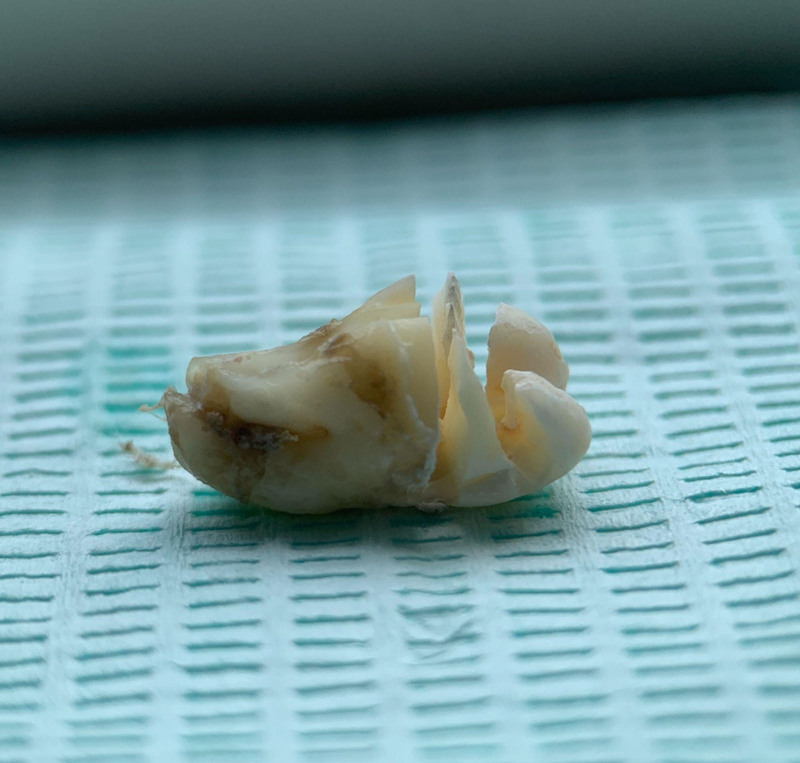
Buccolingual sectioning of the crown and root with elevator insertion for separation, followed by mesiodistal lingual sectioning of crown and root with elevator insertion for separation.

**Figure 7.**
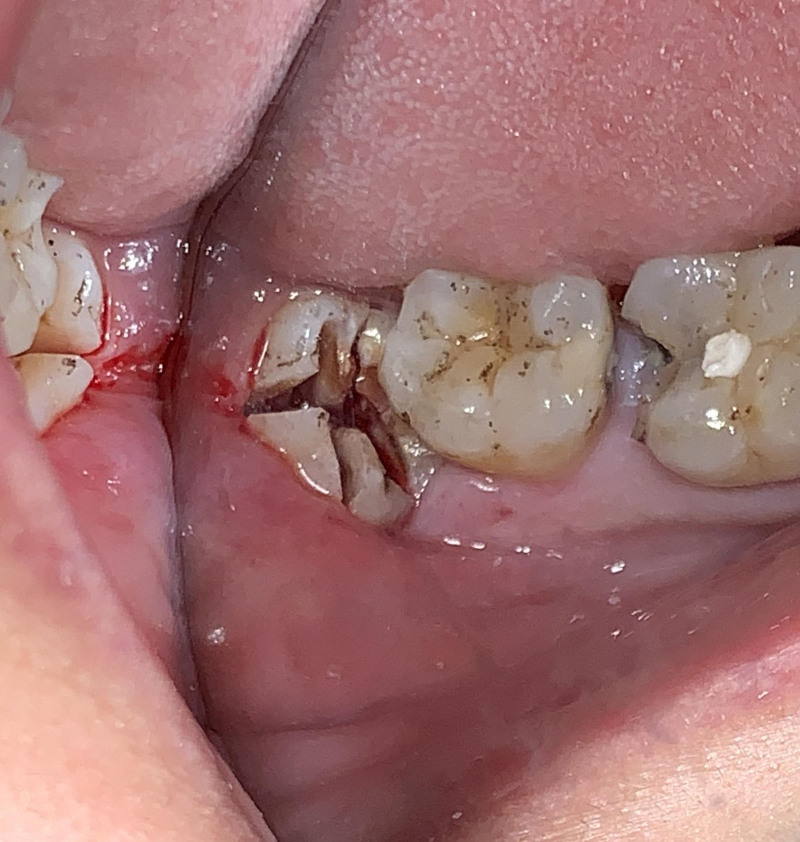
After all 6 parts of the tooth are separated, repeatedly irrigate to remove surface debris.

**Figure 8.**
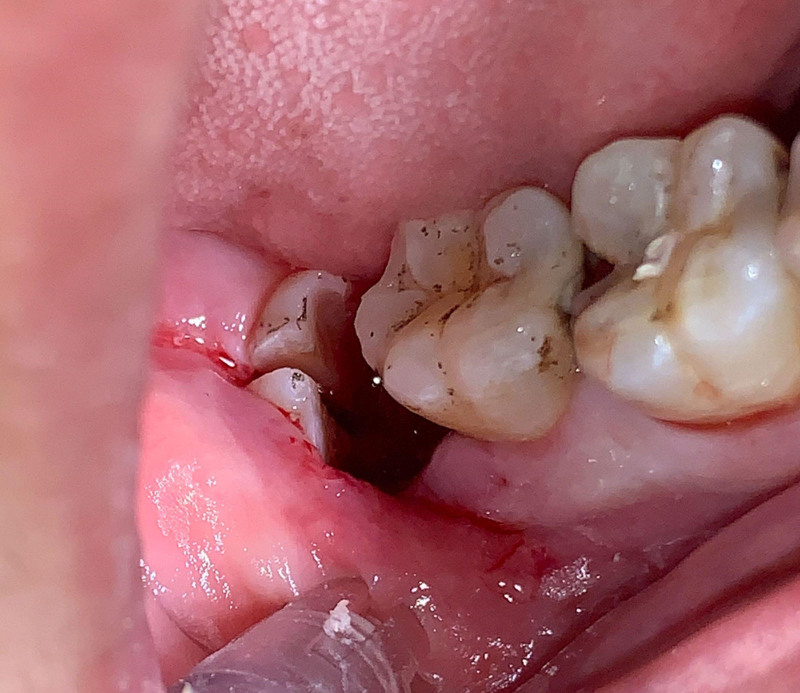
After completely removing the 4 sections of the crown, irrigate again.

**Figure 9.**
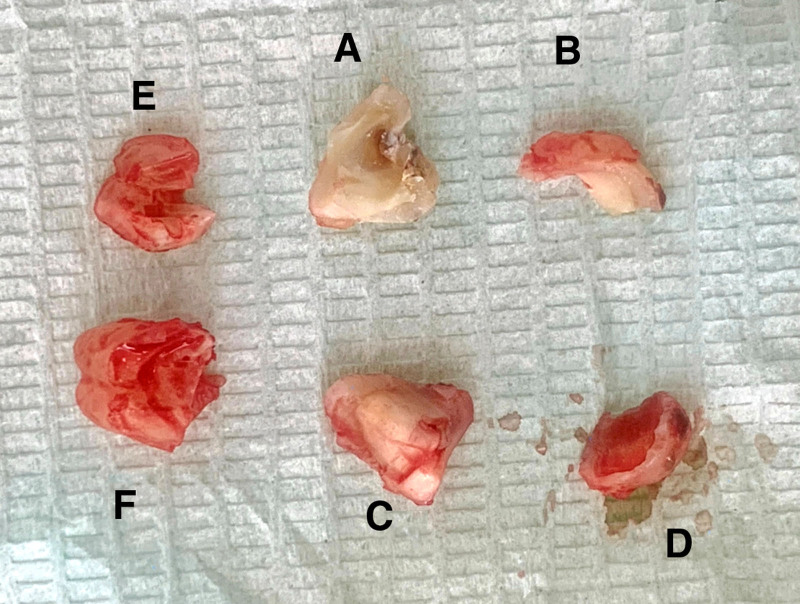
Exhibition of the 6 separated tooth sections.

- Ensure satisfactory anesthesia to achieve pain control and patient comfort.
- Locating the anatomical position of the crown:
- Use CBCT imaging to determine the crown-root junction of the mandibular horizontally impacted tooth (MHIT).
- Before crown sectioning, appropriately separate the surface soft tissue (usually without a flap or only cutting 2 to 3 mm beyond the cementoenamel junction).
- Precise localization of this position is critical, particularly to important adjacent anatomical structures, such as the inferior alveolar nerve.
- Cutting procedure:
- Using a micro-power system with a 45-degree angled high-speed turbine handpiece, perform a buccolingual cut from approximately 2 mm above the cementoenamel junction, passing through the pulp chamber and extending towards the root bifurcation. The cutting range should exceed 2/3 of the buccolingual diameter (Figs. 2–4).
- Follow the length indications of the extraction but accurately control the cutting depth, avoiding cutting into the underlying tooth structure. Ensuring the correct depth prevents unnecessary damage to surrounding structures.
- Insert the elevator into the cut groove and gently elevate to separate the crown and root.
- Perform a vertical cut from the center of the crown downwards, exceeding 2/3 of the vertical height of the crown. Due to limited visibility and operational angles, switch to using the left hand to elevate and separate the crown into buccal and lingual halves (Figs. 4–6).
- Make additional cuts centrally in the buccal and lingual sections, and after elevation, separate the crown into 4 parts. Once the resistance is relieved, sequentially remove the parts (Figs. 2, 5–7).
- Thoroughly rinse with saline to remove tooth fragments, food debris, detached calculus, and infected granulation tissue.
- Examine the root section and perform a vertical cut from approximately 2/5 of the lingual side to a depth of about 3 mm. Elevate to remove the lingual portion (Fig. 8).
- Insert the elevator into the periodontal space from the buccal side, rotating the buccal portion of the root towards the lingual side for extraction.
- If there is inter-radicular resistance, perform horizontal cuts to section the roots and then remove them in parts (Fig. 9).

##### 2.3.3.3. Technical details

- Throughout the procedure, the cutting is primarily confined to the internal tooth structure. Separation relies on the tooth structure itself as support, utilizing the spaces created by cutting between tooth structures and the periodontal space. This method effectively avoids repeated hammering and elevating against bone tissue and adjacent teeth.
- Perform an initial buccolingual “I” shaped cut, followed by a “T” shaped cut, and a final horizontal cut at the root section. Ultimately, use a “半” shaped section to divide the tooth into 6 parts. This systematic approach to sectioning minimizes trauma.

##### 2.3.3.4. Justification of method selection

- Use of CBCT imaging: CBCT provides a detailed 3-dimensional view of the impacted tooth and surrounding structures, allowing precise surgical planning and reducing the risk of complications.
- Micro-power system: The adoption of a micro-power system for the “半” shaped crown sectioning method is based on its ability to offer high-speed, precise cutting with minimal tissue trauma, aligning with the goal of minimally invasive surgery.
- Sequential sectioning technique: This method reduces mechanical resistance, making the extraction process more efficient and less traumatic, thereby enhancing patient recovery.
- “半” Shaped crown sectioning method: This novel technique introduces a systematic approach to sectioning the crown and roots, aiming to reduce operation time, trauma, and postoperative pain compared to traditional methods.
- Minimally invasive approach: The “半” shaped crown sectioning method aligns with the principles of minimally invasive surgery, promoting faster healing and better postoperative outcomes.

### 2.4. Observation indicators

- **Operation time:** Record the duration from gingival separation to the patient leaving the dental chair, measured in minutes.
- **Alveolar damage:** Observe and score the damage to the buccal, lingual, mesial, and alveolar socket base. Each damaged area scores 1 point, with higher scores indicating more severe alveolar damage.
- **Postoperative pain:** Assess postoperative pain using the international visual analog scale (VAS), with scores ranging from 0 to 10. Higher scores indicate more severe pain.
- **Postoperative mouth opening degree:** Record the distance between the upper and lower central incisor edges at the maximum mouth opening.

#### 2.4.1. Scoring

- ≥2.5 cm (unrestricted) = 0 points.
- 2.0 cm ≤ distance < 2.5 cm (mild restriction) = 1 point.
- cm ≤ distance < 2.0 cm (moderate restriction) = 2 points.
- <1.0 cm (severe restriction) = 3 points.
- Higher scores indicate greater restriction.

#### 2.4.2. Wound healing condition

##### 2.4.2.1. Soft tissue healing

- Complete coverage of the bone surface with no chronic inflammation = 0 points.
- Incomplete coverage with no chronic inflammation = 1 point.
- Incomplete coverage with chronic inflammation = 2 points.
- Higher scores indicate poorer healing.

##### 2.4.2.2. Bone tissue imaging

- Clear alveolar socket contour with lower bone density inside the extraction socket compared to surrounding bone = 1 point.
- Unclear contour with similar bone density inside the socket to surrounding bone = 2 points.
- Unclear contour with inconsistent bone density = 3 points.
- Presence of necrotic bone = 4 points.
- Higher scores indicate poorer bone tissue imaging results.

### 2.4. Statistical methods

Data were analyzed using SPSS 22.0 software (Chicago). Normally distributed measurement data were expressed as mean ± standard deviation. Independent samples test was used for intergroup comparisons of normally distributed data, and the Mann–Whitney *U* test was used for non-normally distributed data. *P*-value of <.05 was considered statistically significant.

## 3. Results

### 3.1. Comparison of operation time, alveolar damage, and postoperative pain index between the 2 groups

The study group exhibited significantly shorter surgery times, less alveolar damage, and lower postoperative pain compared to the control group. Specifically, the mean surgery time for the study group was 20.46 ± 3.08 minutes, while the control group required 32.11 ± 4.12 minutes (*t* = 20.486, *P* < .000). Alveolar damage scores were 2.13 ± 0.22 in the study group and 3.33 ± 0.43 in the control group (*t* = 11.564, *P* < .000). The VAS scores were 3.59 ± 1.41 for the study group and 4.96 ± 1.35 for the control group (*t* = 6.364, *P* < .000) (Table 1).

**Table 1:** Comparison of surgery time, alveolar damage, and postoperative pain index between 2 groups of patients (X ± SD).

Group	Cases	Surgery time (min)	Alveolar damage (score)	VAS (score)
Study group	120	20.46 ± 3.08	2.13 ± 0.22	3.59 ± 1.41
Control group	120	32.11 ± 4.12	3.33 ± 0.43	4.96 ± 1.35
*t*		20.486	11.564	6.364
*P*		.000	.000	.000

VAS = visual analog scale.

### 3.2. Comparison of postoperative mouth opening degree between the 2 groups

Postoperative mouth opening was significantly better in the study group. In the study group, 40.83% of patients had no restriction (score 0), 42.5% had mild restriction (score 1), 15.83% had moderate restriction (score 2), and only 0.83% had severe restriction (score 3). In contrast, the control group had 26.67% with no restriction, 37.5% with mild restriction, 27.5% with moderate restriction, and 8.33% with severe restriction (*Z* = 3.689, *P* < .000) (Table 2).

**Table 2:** Comparison of postoperative mouth opening between 2 groups of patients (n, [%]).

Group	Cases	Score			
		0	1	2	3
Study group	120	49 (40.83)	51 (42.5)	19 (15.83)	1 (0.83)
Control group	120	32 (26.67)	45 (37.5)	33 (27.5)	10 (8.33)
*Z*		3.689			
*P*		.000			

### 3.3. Comparison of postoperative soft tissue healing between the 2 groups

Soft tissue healing was significantly better in the study group. The study group had 33.33% of patients with complete soft tissue coverage (score 0), 58.33% with incomplete coverage without chronic inflammation (score 1), and 8.33% with incomplete coverage with chronic inflammation (score 2). The control group had 23.33% with complete coverage, 41.67% with incomplete coverage without inflammation, and 35.00% with incomplete coverage with inflammation (*Z* = 3.480, *P* = .001) (Table 3).

**Table 3:** Comparison of postoperative soft tissue healing between 2 groups of patients (n, [%]).

Group	Cases	Score		
		0	1	2
Study group	120	40 (33.33)	70 (58.33)	10 (8.33)
Control group	120	28 (23.33)	50 (41.67)	42 (35.00)
*Z*		3.480		
*P*		.001		

### 3.4. Comparison of postoperative bone tissue imaging results between the 2 groups

Bone tissue imaging showed significantly better outcomes in the study group. The study group had 40.83% of patients with clear alveolar socket contour and lower bone density inside the extraction socket (score 1), 46.67% with unclear contour but similar bone density (score 2), 11.67% with unclear contour and inconsistent bone density (score 3), and 0.83% with necrotic bone (score 4). The control group had 28.33% with a score of 1, 36.67% with a score of 2, 38.33% with a score of 3, and 13.33% with a score of 4 (*Z* = 3.004, *P* = .003) (Table 4).

**Table 4:** Comparison of bone tissue imaging results between 2 groups of patients (n, [%]).

Group	Cases	Score			
		1	2	3	4
Study group	120	49 (40.83)	56 (46.67)	14 (11.67)	1 (0.83)
Control group	120	34 (28.33)	44 (36.67)	46 (38.33)	16 (13.33)
*Z*		3.004			
*P*		.003			

### 3.5. Additional observations

Demographic parameters, including gender distribution, age, smoking history, and alcohol consumption history, were not significantly different between the 2 groups (*P* > .05). The study group also experienced fewer complications and higher patient satisfaction compared to the control group (*P* < .05) (Table 5).

**Table 5:** Number of patients, gender, age, smoking, and alcohol history.

Parameter	Study group (“半” shaped crown sectioning method)	Control group (ultrasonic instrument cutting method)	*P*-value
Number of patients	120	120	–
Gender distribution (male/female)	62/58	61/59	>.05
Age (yr)	19 to 55 (33.44 ± 5.45)	20 to 56 (34.98 ± 5.55)	>.05
Smoking history	58	53	>.05
Alcohol consumption history	70	78	>.05
Operation time (min)	Shorter	Longer	<.05
Trauma level	Less	More	<.05
Postoperative pain (VAS score)	Lower	Higher	<.05
Healing time (d)	Faster	Slower	<.05
Complications	Fewer	More	<.05
Patient satisfaction (rating)	Higher	Lower	<.05

VAS = visual analog scale.

## 4. Discussion

Mandibular horizontally impacted teeth (MHIT) are a significant cause of distal caries and periodontal damage to second molars. The local blind pocket and the oral hygiene dead angle associated with MHIT contribute to recurrent inflammatory reactions and the spread of infection.^[3]^ The proliferation of bacteria and toxin release in this area can also be sources of systemic diseases. Therefore, timely extraction of MHIT can effectively reduce the risk of oral infections and other oral diseases.^[7]^

The removal of MHIT is particularly challenging due to the varied positions and root morphology of the teeth, constraints imposed by the patient’s oral anatomy, and the posterior location, which limits visibility and operating space. These factors result in prolonged extraction times and intense postoperative pain for patients, making this procedure one of the more difficult operations in oral and maxillofacial surgery.^[8]^ The pressure exerted by these impacted teeth on the anterior second molars creates a tight interproximal relationship in the posterior molar region. Additionally, the mandibular bone, enveloped by a hard external oblique ridge cortex, complicates extraction as the teeth cannot be easily separated from surrounding tissues. This often necessitates the removal of surrounding soft tissue, second molar, peripheral bone walls, and root bone tissue, while also considering the important relationships with the lingual nerve and inferior alveolar nerve.^[9]^

Traditional turbine-driven extraction involves cooling with non-sterile water and cutting the crown to remove the impacted teeth, which is currently the most widely used method.^[10,11]^ However, the lack of a standardized procedure and the need for repeated cutting to overcome crown resistance can result in significant damage to the gingiva and alveolar bone, leading to intense postoperative pain.^[12,13]^ Compared to the traditional turbine method, the “半” shaped crown sectioning method with a micro motor system offers shorter extraction times, less damage to adjacent tissues, and reduced postoperative pain. Its efficacy has been preliminarily recognized in clinical practice.^[14]^

In this study, the application of the “半” shaped crown sectioning method in treating MHIT showed that the surgery duration in the study group was shorter than that in the control group. Bone resistance and adjacent tooth resistance are the main sources of crown resistance in impacted teeth. By eliminating these resistances, the efficiency of impacted tooth extraction is greatly improved. The “半” crown sectioning method divides the large crown into 6 parts, reducing the resistance on the periphery of the crown and avoiding the need to remove adjacent buccal bone tissue. This simpler and more standardized procedure significantly shortens extraction time. The study group also had lower alveolar injury scores compared to the control group, indicating that the “半” shaped crown sectioning method causes less harm to the oral soft tissues.

MHIT are generally located deep distal to the second molars, and their removal may involve surrounding soft tissue entanglement and friction, or even accidental cutting. Poorly designed cutting can lead to root fracture, damage to adjacent teeth, and even tooth loosening and loss. The “半” shaped crown sectioning method ensures the preservation of periodontal soft tissue structure and minimizes damage to the buccal, lingual, and mesial areas. The cutting process targets only the crown, avoiding damage to surrounding blood vessels and nerve tissues, thus offering high safety.

Postoperative VAS scores were lower in the study group than in the control group; the range of mouth opening was greater, and the wound healing was better in the study group, indicating that the “半” shaped crown sectioning method improves patient outcomes, comfort, and postoperative recovery speed. This method uses a high-speed contra-angle turbine in the micromotor system to cut and separate the impacted tooth crown into 6 parts, which are then sequentially removed. This approach effectively avoids damage to surrounding nerves, reduces postoperative pain, and enhances patient recovery.

In summary, the “半” shaped crown sectioning method in the extraction of MHIT reduces the need for buccal alveolar bone removal, shortens surgical time, decreases alveolar damage, alleviates postoperative pain, improves mouth opening, and accelerates wound healing, making it highly valuable for clinical promotion.

## 5. Conclusion

The detailed statistical analysis confirms that the “半” shaped crown sectioning method offers several advantages over the traditional ultrasonic instrument cutting method in the extraction of MHIT. These advantages include shorter operation times, less trauma, lower postoperative pain, faster healing, fewer complications, and higher patient satisfaction. These findings support the clinical adoption and implementation of the “半” shaped crown sectioning method as a preferred approach in dental practice.

## Acknowledgments

The patient in the case had given her informed consent in writing before inclusion in the study. Informed consent was obtained from the patient to publish this case report details.

## Author contributions

**Conceptualization:** Caiwang Chang.

**Methodology:** Juanjuan Gao.

**Project administration:** Caiwang Chang.

**Resources:** Le Yang, Jinhua Huang.

**Software:** Miaomiao Shao, Qingmei Kong.

**Visualization:** Zhibing Meng.

**Writing – original draft:** Tingting Zhang.
